# A Case of Occult Breast Cancer Diagnosed during Immune Checkpoint Inhibitor Treatment for Recurrent Metastatic Lung Cancer

**DOI:** 10.70352/scrj.cr.25-0389

**Published:** 2025-10-15

**Authors:** Mio Makino, Hiroki Kusama, Masaru Hagiwara, Yoshiya Horimoto, Eiichi Sato, Norihiko Ikeda, Takashi Ishikawa

**Affiliations:** 1 Department of Breast Surgical Oncology, Tokyo Medical University, Tokyo, Japan; 2 Department of Surgery, Tokyo Medical University, Tokyo, Japan; 3 Department of Pathology (Medical Research Center), Institute of Medical Science, Tokyo Medical University, Tokyo, Japan

**Keywords:** occult breast cancer, immune checkpoint inhibitor, non-small cell lung cancer, axillary lymphadenopathy, 2nd primary malignancy

## Abstract

**INTRODUCTION:**

Occult breast cancer (OBC) is a rare subtype of breast cancer, typically presenting as axillary lymph node metastasis without an identifiable primary tumor in the breast. Axillary lymphadenopathy requires differential diagnosis, including OBC. However, in patients undergoing treatment for another malignancy, distinguishing OBC from axillary metastasis of the known primary cancer can be challenging. Immune checkpoint inhibitors (ICIs) have extended survival in advanced non-small cell lung cancer (NSCLC), potentially allowing time for 2nd primary cancers to develop and be detected.

**CASE PRESENTATION:**

A 71-year-old woman underwent right upper lobectomy for stage IIIA lung adenocarcinoma. Four months postoperatively, CT revealed a right chest wall mass and right axillary lymphadenopathy, which was interpreted as recurrence. Systemic therapy was administered, and third-line atezolizumab monotherapy led to complete remission of the chest wall mass; however, progressive enlargement of the axillary lymph nodes was subsequently observed. Imaging showed no detectable lesion in the breast, but core needle biopsy of the axillary node revealed metastatic invasive ductal carcinoma, negative for estrogen receptor (ER), progesterone receptor (PR), and human epidermal growth factor receptor 2 (HER2) with a Ki-67 index of 80%. Immunohistochemistry was positive for GATA3 and negative for thyroid transcription factor-1 (TTF-1), consistent with OBC. The patient underwent axillary lymph node dissection, and postoperative observation without additional treatment was selected due to comorbidities. She has remained disease-free for 1 year.

**CONCLUSIONS:**

This case illustrates that axillary lymphadenopathy during treatment for another malignancy may represent a 2nd primary cancer such as OBC. As ICI therapy prolongs survival, clinicians should pay attention for new malignancies, including breast cancer, even in the absence of breast lesions.

## Abbreviations

ALK
: anaplastic lymphoma kinase
EGFR
: epidermal growth factor receptor
ER
: estrogen receptor
GCDFP-15
: gross cystic disease fluid protein-15
H&E
: hematoxylin and eosin
HER2
: human epidermal growth factor receptor 2
ICI
: immune checkpoint inhibitor
MDS
: myelodysplastic syndrome
NSCLC
: non-small cell lung cancer
OBC
: occult breast cancer
PD-L1
: programmed death-ligand 1
PR
: progesterone receptor
TTF-1
: thyroid transcription factor-1

## INTRODUCTION

OBC is a rare subtype of all breast cancers, accounting for approximately 0.1% of them.^1)^ It is characterized by axillary lymph node metastases with no identifiable primary tumor in the breast. Compared with primary breast cancer, OBC has a lower proportion of tumors that express hormone receptors, with only around 40% of cases being ER-positive.^2)^ Even in the absence of breast findings, axillary lymphadenopathy should raise suspicion for OBC. When axillary lymphadenopathy is observed during treatment for other malignancies, it is essential to differentiate between metastasis from the known primary tumor, breast cancer metastasis, OBC, malignant lymphoma, and infection. We report a case in which isolated right axillary lymphadenopathy developed during immunotherapy for stage IV lung cancer, leading to the diagnosis of occult breast cancer.

## CASE PRESENTATION

**Fig. 1:**
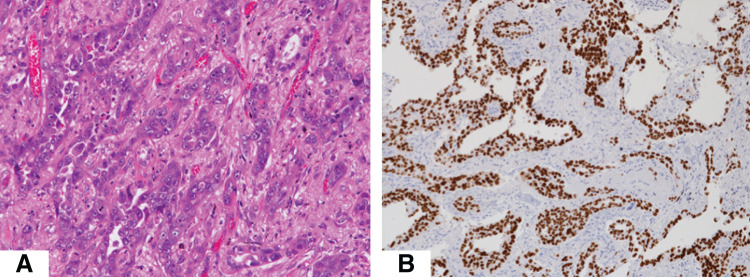
Histological findings of the primary lung cancer lesion (**A**) Hematoxylin and eosin staining shows papillary proliferation of atypical cells. (**B**) Tumor cells were positive for TTF-1 staining. TTF-1, thyroid transcription factor-1

We first describe the clinical course of treatment for primary lung cancer. A 71-year-old patient was found to have a solid tumor 3.5 × 2.2 cm in size in the right upper lobe on CT. Subsequent clinical evaluation led to a definitive diagnosis of primary lung cancer, staged as cT2aN0M0, stage IIB. The patient underwent right upper lobectomy with mediastinal lymph node dissection. The final pathological findings showed papillary adenocarcinoma measuring 3.5 cm in maximal dimension, with metastasis mediastinal lymph node (n = 1/23). The pathological stage was pT2N2M0, stage IIIA. H&E staining and negative TTF-1 immunohistochemistry of the lung tumor are shown in **Fig. 1**.

**Fig. 2:**
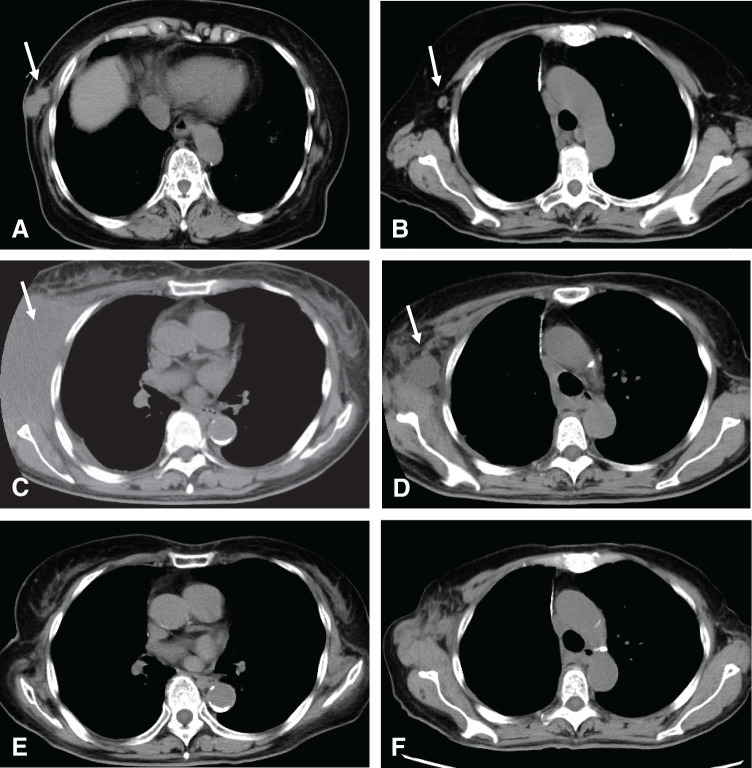
Sequential CT images over time. The left row shows chest wall recurrence (arrows), and the right row shows axillary lymph nodes (arrows). (**A**), (**B**) Findings at recurrence, 4 months postoperatively. (**C**), (**D**) At the time of regrowth of the chest wall tumor and axillary lymph nodes, 16 months after initiation of second line systemic therapy. (**E**), (**F**) At the time of response after 6 months of third line systemic therapy.

Due to the patient's advanced age and suspicion of interstitial pneumonia on preoperative CT, adjuvant therapy was not administered postoperatively. Four months after surgery, a right chest wall mass and right axillary lymphadenopathy was detected on CT (**Fig. 2A**, **2B**). These findings were considered recurrence, and systemic therapy was initiated.

Molecular testing revealed negative results for EGFR mutations and ALK rearrangements, with a PD-L1 tumor proportion score of 60%. Cisplatin, pemetrexed, and pembrolizumab were administered as first-line treatment. After 4 cycles, chest wall mass and axillary lymph node decreased in size. However, 6 months later, CT showed re-enlargement of the chest wall tumor, and second-line treatment with docetaxel and ramucirumab was initiated. Despite temporary treatment interruptions due to adverse events, therapy was continued. Sixteen months after the initiation of second-line therapy, rapid enlargement of the chest wall mass and right axillary lymphadenopathy was observed (**Fig. 2C**, **2D**).

Third-line therapy with atezolizumab monotherapy was initiated. The chest wall mass showed marked reduction, and the axillary lymph nodes also demonstrated slight shrinkage until the 6-month CT scan. Thereafter, the lymph nodes gradually enlarged, and the patient was referred to our department at 23 months after the initiation of third-line therapy (**Fig. 2E**, **2F**).

Subsequently, the chest wall mass remained in complete remission; however, progressive enlargement of the right axillary lymph nodes was observed. Twenty-three months after the third-line therapy, the patient was referred to our department for further evaluation.

**Fig. 3:**
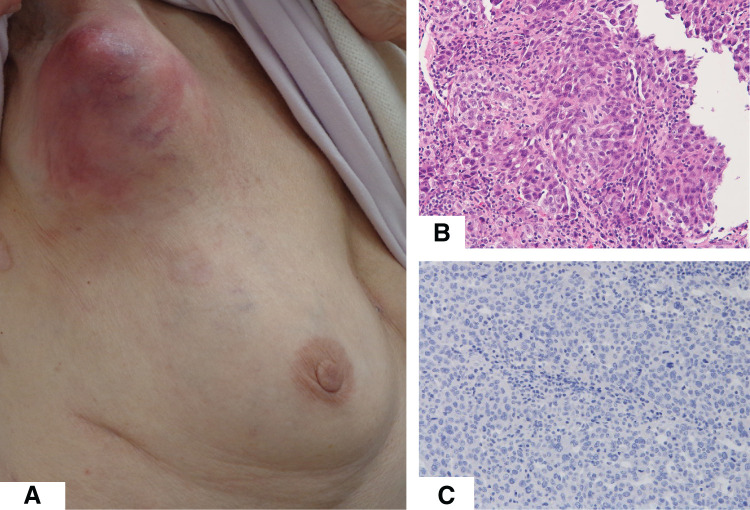
(**A**) An axillary mass with skin erythema. (**B**) H&E staining of axillary lymph node showing proliferation of atypical cells forming solid nests. (**C**) Immunohistochemical staining for TTF-1 was negative in this lesion. H&E, hematoxylin and eosin; TTF-1, thyroid transcription factor-1

On physical examination, an 8-cm mass with overlying skin erythema was palpated in the right axilla (**Fig. 3A**). Mammography and breast ultrasound revealed no detectable lesions in the breast. However, ultrasound demonstrated marked enlargement of the axillary lymph nodes. A core needle biopsy was conducted on the axillary lymph node.

Histopathological analysis revealed complete effacement of the normal lymph node architecture. Nests of atypical cells replaced the native lymphoid tissue (**Fig. 3B**). These findings were morphologically distinct from the previously resected lung cancer, and the tumor cells were negative for TTF-1 (**Fig. 3C**). Based on these findings, the lesion was diagnosed as axillary lymph node metastasis of breast cancer, specifically OBC, classified as cT0N2M0, Stage IIIA.

Immunohistochemistry showed that the tumor cells were negative for ER, PR and HER2, Ki-67 labeling index of 40%.

**Fig. 4:**
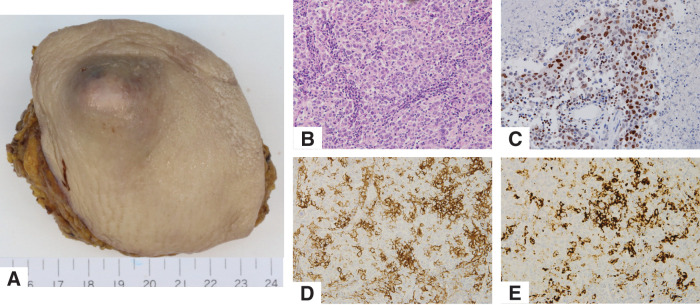
(**A**) Macroscopic findings of axillary lymph node with skin. (**B**) Hematoxylin eosin staining of the tumor. (**C**) Tumor cells were GATA3-positive. (**D**) PD-L1 (22C3). CPS was 30, (**E**) PD-L1 (SP142). Score was IC3 (≥10%). CPS, combined positive score; IC, immune cell; PD-L1, programmed death-ligand 1

We performed a right axillary lymph node dissection with curative intent. Surgical resection included the skin directly overlying the tumor and partial resection of the pectoralis major and minor muscles due to direct tumor invasion. As there was no invasion of the axillary vein and the thoracodorsal artery, vein, and nerve, they were preserved. A total of 14 lymph nodes were removed, 8 from Level I and 6 from Level II. Macroscopic and histologic findings are shown in **Fig. 4A**.

Postoperative pathology revealed metastatic involvement in 2 of the 14 dissected lymph nodes. Histological examination of the axillary lymph node revealed metastatic invasive ductal carcinoma of breast origin, negative for ER, PR, and HER2, Ki-67 labeling index of 80%.

To differentiate from the previously diagnosed lung carcinoma, additional immunohistochemistry was performed. The tumor was positive for GATA3 and negative for GCDFP-15, mammaglobin, and TTF-1—findings consistent with OBC (**Fig. 4B**, **4C**). PD-L1 immunostaining showed high expression with both the 22C3 and SP142 clones (**Fig. 4D**, **4E**).

Postoperative treatment for OBC was not administered due to the presence of interstitial pneumonia which contraindicated radiotherapy and the patient's advanced age and history of recurrent lung cancer. As the chest wall mass from lung cancer remained in complete remission, atezolizumab was also discontinued. The patient is currently under observation without active treatment and has remained recurrence-free for 1 year postoperatively.

## DISCUSSION

We reported a rare case in which OBC was diagnosed in a long-term survivor of metastatic recurrent lung cancer. Right axillary lymphadenopathy was initially treated as a metastasis from lung cancer, but a biopsy confirmed the diagnosis of OBC. Surgical resection was performed, and although the possibility of future distant recurrence of breast cancer or relapse of lung cancer remains, the patient has since been managed with observation alone. This approach significantly reduced the burden of hospital visits and treatment costs, proving to be an effective treatment strategy in this context.

With the advent of immune checkpoint inhibitors (ICIs), the prognosis of advanced and recurrent non-small cell lung cancer (NSCLC) has improved dramatically.^3–5)^ In a subset of patients, a state resembling remission—so-called “tail plateau” effects—have been observed, enabling long-term survival.^6)^ In cases with a short prognosis, breast cancer may have gone undiagnosed and remained clinically insignificant, but with extended survival, the number of diagnoses may gradually increase. In a small-scale, single-institution study, secondary malignancies involving 12 organs were reported in 10 cases among 112 long-term survivors (≥2 years) of advanced NSCLC; no cases of breast cancer were observed.^7)^ By contrast, the Surveillance, Epidemiology, and End Results (SEER) database revealed that among 282486 patients diagnosed to have lung cancer, 654 cases (0.2%) were also diagnosed to have either synchronous or metachronous breast cancer. Of these, 144 cases involved metastatic lung cancer, and 48 patients were diagnosed with breast cancer following the initial lung cancer diagnosis.^8)^ These reports indicate that the incidence of breast cancer after a diagnosis of lung cancer is very rare. However, as cancer therapies continue to advance, it is likely that such cases will increase. In particular, patients achieving long-term survival with ICIs may require routine cancer screening, including breast cancer screening.

In this case, the chest wall mass showed a complete response to atezolizumab monotherapy, and the axillary lymph nodes also initially decreased in size, which prompted suspicion of a new primary malignancy only upon their subsequent re-enlargement. Tumor immunogenicity is a key determinant of ICI efficacy. In general, breast cancer is considered less immunogenic than lung cancer.^9)^ ICI monotherapy for metastatic breast cancer has been evaluated in phase I trial, with modest response rates (0%–9.6%) and progression-free survival of approximately 1.4 months. Although prolonged responses have been reported in cases where shrinkage is achieved,^10)^ combination therapy with chemotherapy is recommended as the standard practice.^11,12)^

In our case, the axillary lymph nodes initially showed shrinkage, suggesting that atezolizumab might have conferred a clinical benefit. In addition, because no other systemic therapies were available for the patient’s lung cancer, atezolizumab was continued despite enlargement of the axillary lymph nodes, resulting in a total treatment period of 23 months. At the time of progression, the axillary lymph node metastasis showed strong PD-L1 expression. However, given its initial response to ICI monotherapy, it is possible that immune resistance, such as effector T cell exhaustion or reestablishment of an immunosuppressive tumor microenvironment,^13)^ developed during the course of treatment. Considering these factors, our case underscores the limitations of ICI monotherapy in breast cancer and the necessity of combination approaches with chemotherapy.

In this case, early surgical intervention was necessary, and contrast-enhanced MRI for breast evaluation was not performed. Based on the clinical course and pathological findings, the diagnosis of OBC was made. However, for similar cases in the future, it is desirable to confirm the presence or absence of the primary lesion using contrast-enhanced MRI.

## CONCLUSIONS

We experienced a case in which OBC was diagnosed during ICI treatment for recurrent metastatic lung cancer. When lymph node enlargement is observed during cancer treatment, it is necessary to consider not only metastasis from the primary malignancy but also the possibility of axillary lymph node metastasis from breast cancer.
